# Artificial Respiration

**Published:** 1857-08

**Authors:** Marshall Hall

**Affiliations:** The Institute of France


### Artificial Respiration.

*The Danger of all Attempts at Artificial Respiration, Except in the Prone Position.*—By Marshall Hall, M. D., F. R. S., &c., of the Institute of France, etc.—

I have shown, in a previous paper, not the inutility only, but the *danger* of the *warm bath* in the treatment of apnœa or asphyxia, except in the *prone* position.

If the asphyxiated patient be moved and placed in the supine position, in which no attempts at artificial respiration can be effectually made, what is the condition of the rima glottidis, or entrance into the windpipe? Is it *free*, so that air may be pressed or drawn into into? And if apparently free, does it remain so at the moment when an effort to force or draw air into it is made?

1. Is the tongue so securely situated, all muscular energy having ceased, as neither to *fall* backwards nor to be *drawn* backwards, and so close or obstruct the orifice and entrance into the windpipe?

2. Is there no accumulation of mucus, or other animal fluid, or of fluids from regurgitation from the stomach, which may also obstruct the glottis? nay, more, which may be forced or drawn into the windpipe, inducing a *second* and fatal suffocation?

No one can say *a priori*, that one or even both of those events may not occur. These are not only possible, but probable—not *only* probable, but inevitable under certain circumstances.

There is one fact of the utmost importance. When, from any circumstances, the nervous and muscular powers are in abeyance, nothing is so common as regurgitation from the stomach, from change of position, compression, &c. Under such circumstances, compression of the sides of the thorax would certainly be apt to produce this effect. Now, in the supine position, the matters so regurgitated would remain in the fauces, obstruct the glottis, or, when the pressure was removed, be drawn into the windpipe.—Leroy's mode of attempting to effect artificial respiration, of which a sketch is given by the Royal Humane Society in its reports, is utterly ineffectual: but if effectual, would be replete with danger. The only certain safeguard against such a fatal accident is—*the prone position.* In this position, the tongue tends to fall forwards, and all fluids flow from the fauces and the mouth, or are expelled by the first induced expiration.

All this is reasonable, *a priori.* But we must not rest here.—Our appeal must be to *facts,* not to mere notions. The facts must be ascertained by careful examination of the dead subject.

1. What is the position of the *tongue* when the body has been roughly moved about and laid in the supine position, all cadaveric rigidity of the parts being overcome by previous movement of this organ backwards and forwards ?

2. What is the further position of the tongue in the supine position, at the moment of attempted inspiration, first, by means of the bellows, or, secondly, by the removal of the pressure on the ribs or sternum, and the consequent dilation of the thorax ?

These facts may be ascertained by removing the tissues on one side of the neck, so as to give a lateral view of the tongue, glottis, epiglottis, and pharynx, and by replacing them by a portion of transparent glass of the proper size and form, properly placed and carefully maintained in its position.

The first part of this examination has been already made :—The subject being placed in the supine position, and the lateral parts of the neck being removed, so as to admit of observing the relative position of the internal organs—the tongue, the glottis, the epiglottis, the pharynx—it was seen that obstruction to the entrance of air actually *did* take place.

I now propose to place a piece of transparent glass so as accurately to close the cavity and allow of the observation, first, of the effect of position, the supine and the prone comparatively, and then of any attempt to induce *inspiration.*

A similar examination of this internal in reference to fluids present in it (and we never can know when such fluids are present) is unnecessary; fluids will gravitate to the lowest parts of a cavity, and will be drawn into an open orifice, such as the glottis, under the influence of air forced or inhaled into it. And such an event not only renders all attempts at *in*spiration nugatory, but induces a permanent because material obstruction of the entrance in the windpipe.

In confirmation of these views I again appeal to experimental *facts* :—

"The following experiment has been repeated *many times,* and has been witnessed by George Webster, jun., Esq., of Dulwich; Mr. Williams, Superintendent of the Royal Humane Society, Hyde-Park; and other gentlemen:

"The dead subject being placed in the *supine* position, and pressure made on the sternum and ribs, a little gurgling was heard in the throat; but the pressure being removed, there was no evidence of *in*spiration."

Now let us contrast with these abortive attempts to induce artificial *in*spiration in the *supine* position, the beautiful and life-giving results—*in*spiration and *ex*piration—of alternate rotation from the *prone* position and repronation. I continue the quotation:

"The subject being then turned into the *prone* position, and pressure being made on the spine and ribs, and removed as before, there were free expiration and *in*spiration."

Far more marked is the effect of pronation and rotation:

"The subject was turned into the prone position: considerable expiration took place, which was much augmented by pressure of the hands on the back. On removing this pressure a little inspiration took place. The body being then rotated on the right side, considerable inspiration again took place, whilst moving through one fourth of a circle; on continuing the rotation, inspiration continued until the shoulder was half-way between the lateral position and the table, when it ceased."

These are the original experiments. They are extracted from a little pamphlet entitled "Abstracts of an Investigation into Asphyxia," &c., and now out of print.

I conclude by observing that the principle of *prone respiration* is of such importance as to demand a new designation to impress it on the attention and the memory: I propose to term it PRENOPNŒA.

---

The number of cases of apnœa and asphyxia, the effect of *chloroform*, which have been arrested by the "Ready Method," and of which I have received authentic details, now amount to *three*. The last of these was communicated to me by an eye-witness of the operation, which consisted in tenotomy in a little boy, aged four, under influence of the anæsthetic; suddenly the child turned pale and ceased to breathe, and looked as if it were dead. Cold water was dashed on the face, and other ordinary measures were adopted utterly in vain. The Ready Method was now instantly adopted and efficiently applied: after the first inspiration produced by rotation after pronation with pressure, the mouth was observed to open and air to be inspired; the movements were repeated, physiological respiration commenced, the little boy cried, and all was safe.

I conclude the momentous subject by several *aphorisms* in regard to the treatment of asphyxia:—

1. The effects of suspended respiration can only be removed by the renewal of respiration.

2. Artificial respiration can only be certainly, effectually, and safely performed in the PRONE position; for,

3. In the *supine* position the larynx is apt to be obstructed by the falling back of the tongue and epiglottis, or by the accumulation of fluids already in the mouth or regurgitated from the stomach.

4. These fluids may be fatally inhaled into the windpipe when *in*spiration is mechanically effected.

5. All other measures are subsidiary, even the rubbing the limbs with pressure upwards; and all which exclude respiration are *ipso facto*, destructive; the *warm bath* is of *doubly fatal* tendency,—first, by excluding pronation and rotation, and secondly, by promoting the formation and the circulation of the blood-poison—carbonic acid.—[London Lancet.

